# Overproduction of Sch9 leads to its aggregation and cell elongation in *Saccharomyces cerevisiae*

**DOI:** 10.1371/journal.pone.0193726

**Published:** 2018-03-01

**Authors:** Polina Drozdova, Polina Lipaeva, Tatyana Rogoza, Galina Zhouravleva, Stanislav Bondarev

**Affiliations:** 1 Dept. of Genetics and Biotechnology, Saint Petersburg State University, St. Petersburg, Russia; 2 Vavilov Institute of General Genetics, St. Petersburg Branch, Russian Academy of Sciences, St. Petersburg, Russia; 3 The Laboratory of Amyloid Biology, Saint Petersburg State University, St. Petersburg, Russia; Texas A&M University College Station, UNITED STATES

## Abstract

The Sch9 kinase of *Saccharomyces cerevisiae* is one of the major TOR pathway effectors and regulates diverse processes in the cell. Sch9 belongs to the AGC kinase family. In human, amplification of AGC kinase genes is connected with cancer. However, not much is known about the effects of Sch9 overproduction in yeast cells. To fill this gap, we developed a model system to monitor subcellular location and aggregation state of overproduced Sch9 or its regions fused to a fluorescent protein. With this system, we showed that Sch9-YFP forms detergent-resistant aggregates, and multiple protein regions are responsible for this. This finding corroborated the fact that Sch9-YFP is visualized as various fluorescent foci. In addition, we found that Sch9 overproduction caused cell elongation, and this effect was determined by its C-terminal region containing kinase domains. The constructs we present can be exploited to create superior yeast-based model systems to study processes behind kinase overproduction in cancers.

## Introduction

The Sch9 kinase of *Saccharomyces cerevisiae* has a plethora of functions. Sch9 is one of the major TOR effectors [1]. It takes part in regulation of protein synthesis in response to nutrient availability [1] and cell cycle progression [2]. Sch9 is also linked to such important processes as aging (as deletion of *SCH9* promotes longevity [3]) and maintenance of genome stability (as stable tetraploid clonal populations were characterized by increased Sch9 activity [4]). Strains deleted for *SCH9* are characterized by overall growth defect, which is expressed as significantly decreased cell size and growth rate [5]. However, such strains possess increased thermotolerance, chronological [3] and replicative [6] lifespan. This effect may be explained by constitutively active oxidative stress response system [7] preventing accumulation of age-related mutations [8], perturbed sphingolipid levels [9] or, more likely, a combined effect of these factors.

Sch9 belongs to the AGC kinase family [10]. The AGC (the abbreviation stands for protein kinases A, G and C) kinases are widespread in eukaryotes including both the animal and fungal kingdoms [11]. In *S. cerevisiae* the AGC group contains 17 members, which the Ypk1 and Ypk2 are the closest relatives to Sch9 [10]. Together, the corresponding genes form a co-orthologous group to PKB/SGK kinase genes in mammals, and all members of this group arise from several consequent duplications of a single sequence in the fungal and animal ancestor [12]. In mammals, the PKB and SGK kinases together with RSK and S6K comprise a subfamily of AGC kinases, activated by phosphorylation, members of which are implicated in different diseases including cancer [11]. RSK2 gene overexpression was detected in skin cancer tissues [13]. Amplifications of genome fragments increasing production of PKB/AKT kinases were found in carcinomas of the stomach, ovary, pancreas, and breast [14]. Some data were received about the role of SGK kinase activation in cancer [15, 16], however, S6K is the most extensively studied AGC member. Mammals contain two homologous S6Ks, S6K1 and S6K2, functions of which overlap only partially [17, 18]. The chromosomal region including the gene for S6K1 is amplified in different breast cancer cell lines and in 10-30% of primary tumors [19–21] and was determined as prognostic of metastatic capacity of human breast cancer [22]. At the same time, overexpression of either S6K1 or S6K2 correlates with worse prognosis in breast cancer [23]. Taken together, these data show that protein kinases of the AGC family constitute an important target for cancer therapy, and thus studies in the yeast model might provide important insight into the mechanisms of their functioning.

Although *SCH9* encodes one of the best studied AGC kinases in yeast, the effects of its overexpression have not been studied in detail. Overexpression of *SCH9* is neither lethal nor significantly toxic for the yeast cell [2], even though it causes heat sensitivity [24] and slightly increases the rate of age-dependent mutations [8]. However, evidence on Sch9 overproduction effects is scarce and does not significantly extend beyond the phenotypes listed above.

## Results

### Sch9-YFP is functional and not toxic when overproduced

**Fig 1:**
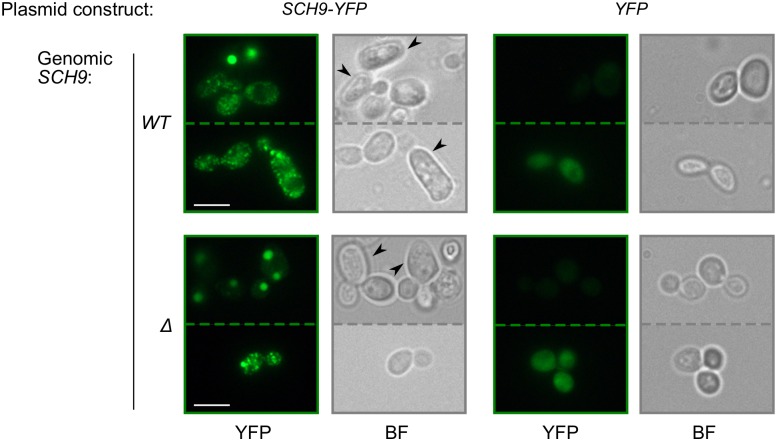
Overproduced Sch9-YFP forms distinct fluorescent foci. Scale bar indicates 5 μm. BF, bright-field microscopy. Dashed lines separated different fields of view.*WT* and *Δ* designate BY4742 and *sch9Δ*-BY4741 strains, respectively. Arrowheads mark elongated cells.

To study effects of *SCH9* overexpression and localization of the corresponding protein we made a plasmid construct for production of Sch9 fused with YFP, under control of strong constitutive GPD (*TDH3*) promoter (p426GPD-SCH9YFP). The control construct (p426GPD-YFP) was identical to the experimental one but did not contain the *SCH9* gene sequence. Then we transformed a wild-type strain (BY4742) and a similar strain deleted for *SCH9* (*sch9Δ*-BY4741) with these constructs and confirmed that full-length Sch9-YFP was produced in both cases (S1A Fig). Moreover, we tested whether the overproduced protein could be phosphorylated and found that at least a fraction of Sch9-YFP had phosphorylated Thr^737^ residue; importantly, Sch9-YFP overproduction did not abolish phosphorylation of the native (genome-encoded) Sch9 (S1B Fig). *SCH9-YFP* overexpression was not toxic in either strain (S1C Fig), which corresponds well to the known data [2]. Interestingly, *sch9Δ*-BY4741 colonies overproducing *SCH9* still did not grow as well as the *SCH9* BY4742 colonies (S1C Fig). It means that these strains have growth differences, and thereafter will only be compared to the corresponding internal control. We also observed that *SCH9* overexpression caused elongation of cells (Fig 1); this fact will be described in detail below.

To check whether the C-terminal YFP tag disturbs functioning of Sch9, we transformed the strain deleted for *SCH9* with a centromeric plasmid bearing *SCH9* driven by its own promoter, analogous construct with *SCH9-YFP* and the corresponding empty vector. Then we compared growth of the transformants on medium containing galactose and raffinose as carbon sources (S1D Fig), as *SCH9* deletion had been found to impede growth on galactose/raffinose-containing media [1]. Indeed, we found that cells with *SCH9* grew better on this medium than the empty vector control (S1D Fig, compare lanes 1 and 2 on galactose/raffinose medium). Importantly, the Sch9-YFP construct was indistinguishable from the one with untagged Sch9 (compare lanes 2 and 3). These data suggest that the YFP tag does not interfere with normal Sch9 functioning.

In addition, we tested whether overproduced Sch9-YFP compensated for this galactose utilization defect and found that it acted in the same way as single-copy *SCH9-YFP*, *i. e.* improved growth on galactose/raffinose-containing medium. Interestingly, we also noticed that the strains overproducing Sch9-YFP grew slower on SC-Ura, as after three days of incubation they formed smaller colonies, but after six days there was almost no difference (S1D Fig). Thus, overproduced Sch9-YFP is at least partially functional (it can be phosphorylated and compensates for the growth defect of the *SCH9*-deleted strain); it is not toxic but decreases cell division rate.

### Overproduced Sch9-YFP forms fluorescent foci and SDS-resistant aggregates

The Sch9-YFP fusion allowed us to monitor subcellular localization of this protein with fluorescent microscopy. Dissimilar to overproduced YFP, which showed the expected diffuse fluorescence (Fig 1, right) and served as a negative control for protein aggregation, overproduced Sch9-YFP formed distinct fluorescent foci, either one or multiple foci per cell (Fig 1, left). We wondered what can determine the fluorescence pattern in particular cells. Importantly, diversity of fluorescent patterns in BY4742 and *sch9Δ*-BY4741 strains was very similar (Fig 1), and for subsequent experiments we preferentially used the *SCH9* strain (BY4742) because of its higher growth rate.

**Fig 2:**
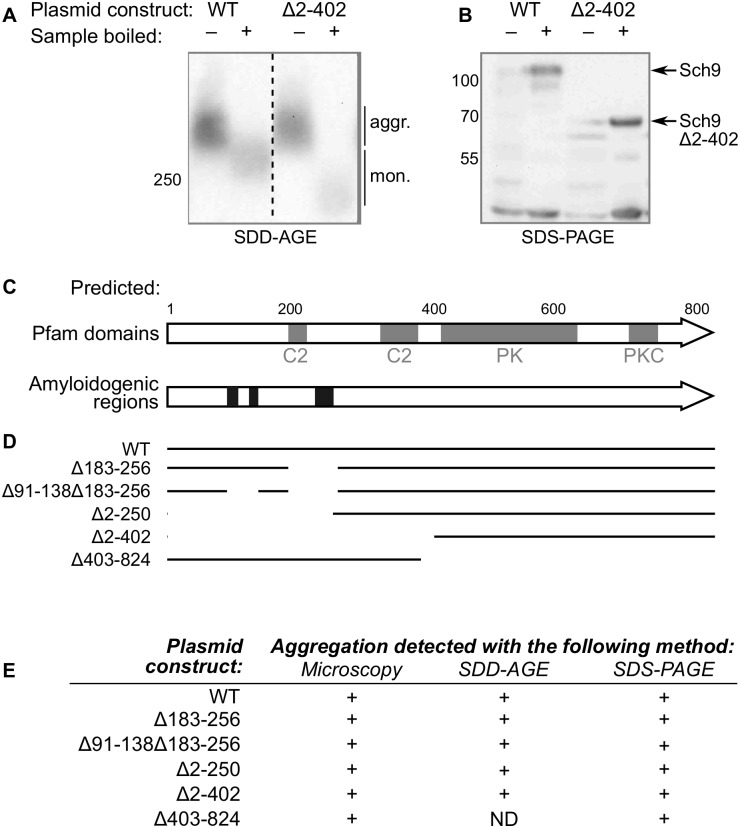
Overproduced Sch9-YFP forms SDS-resistant aggregates. A: Western blotting after SDD-AGE analysis of representative constructs (shown below). Anti-(CGY)FP antibody was used. B: Western blotting after SDS-PAGE analysis. The same lysates as in A were used. Anti-732-743-Sch9-antibody was used. Dashed line separates different lanes from the same blot. Numbers to the left of blots correspond to the molecular weights (kDa) of protein ladder. aggr., aggregated fraction; mon., monomeric fraction. C: Schematic of the Sch9 domain structure according to the *Saccharomyces* genome database (https://www.yeastgenome.org/locus/S000001248) and amyloidogenic regions were predicted using ArchCandy [28] (see Materials and methods for details). PK, protein kinase domain; PKC, protein kinase C domain; C2, domain involved in membrane contact. D: Sch9 deletion constructs used in this work. E: Summary of amyloidogenicity checks for the deletion constructs shown in (C). For detail see S2 Fig.

The Sch9 protein was listed in several screens for potentially amyloidogenic proteins [25, 26]; however, its amyloidogenic properties have not been closely examined. We hypothesized that fluorescent foci visible in the cells may correspond to amyloid aggregates. To check this hypothesis, we performed an SDD-AGE analysis, a method routinely used for analysis of detergent-resistant aggregates [27], and found that overproduced Sch9-YFP indeed formed detergent-resistant aggregates, which dissolved upon boiling with SDS (Fig 2A). This result was further confirmed with SDS-PAGE: only minor fraction of Sch9 was present on lanes with unboiled samples (Fig 2B).

### Different Sch9 domains determine the pattern of its aggregation

Sch9 is enriched with asparagine residues. This feature is typical for yeast aggregation-prone proteins. To determine specific region(s) that might be responsible for aggregation, we chose ArchCandy [28] among several available software tools for amyloidogenicity prediction [29]. Among a variety of different analogs we chose this due to accumulating evidence of its accuracy [28, 30, 31]. This analysis returned several regions in the N-terminal part of the protein (Fig 2C). At the same time, the kinase domains are located in the C-terminal part of the protein. Finally, there are two distinct C2 domains (Fig 2C), which are responsible for membrane binding [32].

We made several deletion constructs removing: the potentially amyloidogenic region with the highest cumulative ArchCandy score (Δ183-256), all regions predicted by ArchCandy (Δ91-138Δ183-256), the N-terminal of the protein until the end of the last of these regions (Δ2-250), the N-terminal part of the protein up to the beginning of the protein kinase domain (Δ2-402), and a reciprocal construct lacking the kinase domains (Δ403-824) (Fig 2D). Each construct was checked with sequencing and SDS-PAGE with Western blotting for production of proteins of expected weights (S2A Fig). Then we tested aggregation with simultaneous analysis with SDD-AGE and SDS-PAGE. In each case, the proteins aggregated, even though in the case of Δ403-824 we could not detect signal on SDD-AGE (Fig 2E, S2B Fig), probably because of low amount of the target protein in cell lysates as judged by SDS-PAGE results (S2B Fig).

Interestingly, even the construct with the longest N-terminal stretch deleted (Δ2-402) formed aggregates indistinguishable from those formed by the full-length protein (Fig 2A, S2B Fig). As both this construct (Δ2-402) and the reciprocal deletion construct (Δ403-824) formed aggregates, as well as all other truncated variants of Sch9, we conclude that this protein contains multiple determinants of aggregation. Nevertheless, we observed changes in aggregates morphology upon overexpression of different constructs. Lack of amino acids 2-250 preserved formation of small foci, while the absence of region 183-256, and potentially 91-38, led to amorphous shape of big aggregates, which are almost spheric in case of wild type.

#### The formation of foci is not connected with the [*PIN*^+^] prion, the IPOD compartment or vacuole

As we observed a number of fluorescent phenotypes (cells with different combinations of large and small dots), we wondered what could determine the observed intracellular location of Sch9 and its aggregation.

First, we posed a question whether Sch9-YFP interacts with the Rnq1 amyloid aggregates. The prion form of the Rnq1 protein, the [*PIN*^+^] (or [*RNQ*^+^]), is present in many wild and laboratory strains [33, 34], particularly in the BY4741 strain [35], which is closely related to the BY4742 strain we used [36]. We obtained a [*pin*^−^] derivative of the BY4742 strain by passaging the strain on guanidinium chloride-containing medium [35]. The different [*PIN*^+^] status of each strain was verified by decoration of the [*PIN*^+^] aggregates with Rnq1-GFP (S3 Fig, upper row). The [*PIN*^+^] and [*pin*^−^] strains showed similar Sch9-YFP fluorescence patterns (S3 Fig, lower row). At least 250 cells for each condition were quantified, and the distributions of cells with different fluorescent patterns were not significantly different (data not shown). Thus, we conclude that the aggregation of Sch9-YFP is independent of [*PIN*^+^].

**Fig 3:**
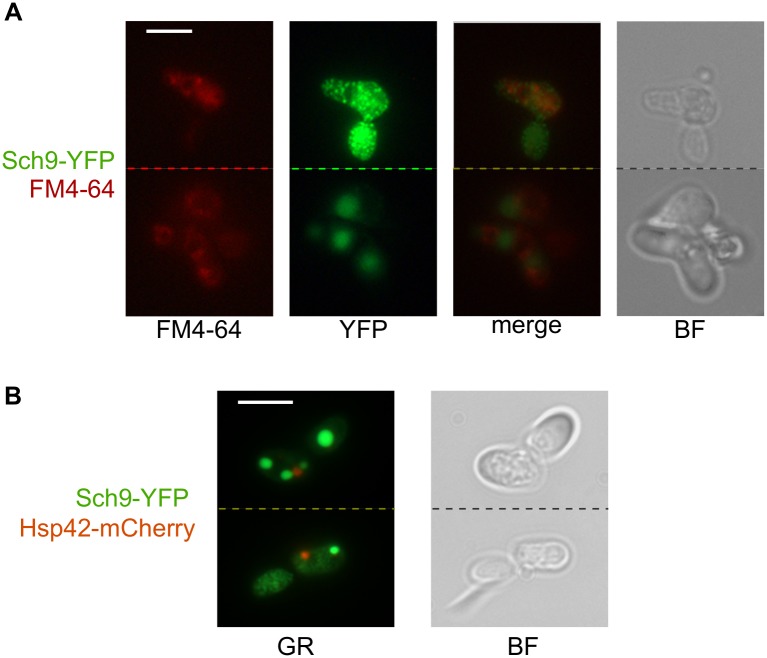
Large Sch9-YFP foci colocalize with neither vacuolar membranes nor IPOD. A: Vacuolar membranes were stained with FM4-64, and these images were merged computationally to study relative location of the stained vacuolar membranes and YFP. B: Co-localization of the Sch9-YFP and Hsp42-mCherry constructs were visualized with the 74 HE GFP+mRFP shift free filter (GR) to observe both proteins at the same time. The scale bar corresponds to 5 μm. BF, bright-field microscopy. Dashed lines separated different fields of view.

Second, Sch9 is known to at least partially reside in the vacuolar membrane [2, 37]. To test whether Sch9-YFP foci might correspond to vacuoles, we used FM4-64, or SynaptoRed C2, to stain vacuolar membranes, and did not see clear colocalization; instead, Sch9-YFP foci were visualized close to the vacuole, but not inside this organelle (Fig 3A). So, our data strongly suggest that even if some of the Sch9-YFP fusion protein is localized in the vacuolar membrane, this fact cannot explain the fluorescence patterns.

Finally, many aggregation-prone proteins are stored in the IPOD compartment localized near the vacuole [38]. Using co-overproduction of Sch9-YFP and Hsp42-mCherry (a marker of IPOD [39]), we checked whether large Sch9-YFP foci might correspond to IPOD. This hypothesis also proved wrong, as in absolute majority (99 out of 100 cells analyzed) Hsp42-mCherry and Sch9-YFP foci did not overlap. A typical example is shown at Fig 3B.

### Overproduction of Sch9 leads to cell elongation, and the C-terminal part of the protein is responsible for this effect

**Fig 4:**
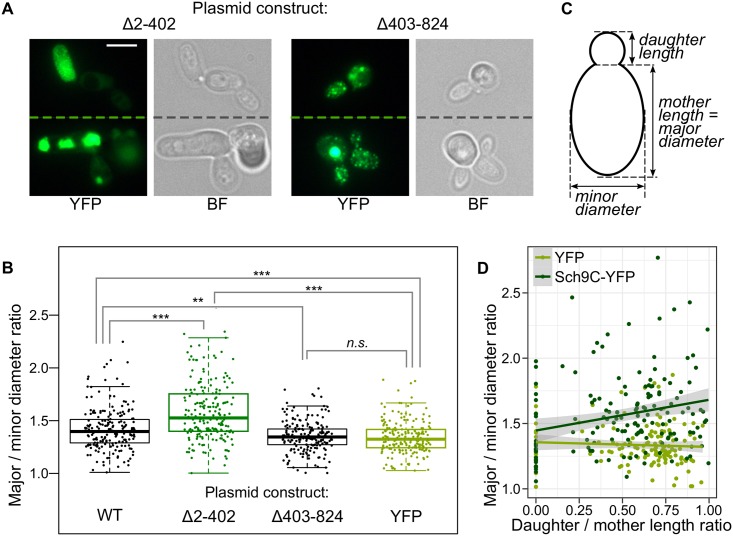
The C- and N-terminal regions of the Sch9 proteins play different roles in its aggregation and phenotype. A: Representative microphotographs of cells overproducing the indicated constructs. Dashed lines separated different fields of view. The scale bar corresponds to 5 μm. BF, bright-field microscopy. B: Box plot summarizing major / minor cell diameter ratios of 200 cells with each construct shown below the graph. Each dot corresponds to individual cell, the central line is the median, box edges show the interquartile range, and whisker length correspond to maximum or minimal values within 1.5 interquartile ranges up and down from the box. **, p < 0.01; ***, p < 0.001; *n.s.*, p > 0.05. Mann-Whitney test with Holm correction was used. C: Schematic of measurements producing values in panels B and D. D: Scatterplot visualizing the relationship between relative bud length and major / minor mother diameter ratio (the vertical axis is the same as in panel B). The line shows predicted values according to a linear regression model; 95% confidence intervals are shown. Bud size, which was determined as the ratio of lengths of daughter and mother cells and, can be used to roughly estimate cell cycle stage [40].

We noticed that BY4742 cells overproducing Sch9-YFP were elongated (Fig 1). To find out which region of the protein was responsible for this effect, we compared form of the cells overproducing either the N-terminal or the C-terminal halves of the protein and found that the cells overproducing the C-terminal region (Sch9Δ2-402) were elongated, while those overproducing the N-terminal region (Sch9Δ403-824) were not (Fig 4A). Interestingly, the fluorescent phenotypes of cells overproducing these constructs were also different: while the C-terminal part of the protein formed either very small dots or large dots (the latter often had irregular shape), the N-terminal part mostly formed clearly visible round dots of varying size (Fig 4A).

The elongation of cells seemed even more prominent in the case of the C-terminal part (Δ2-402) than in the case of the full-length protein (WT) (Fig 4A *cf.* Fig 1). To test this effect statistically, we quantified the ratio of major and minor ellipse diameters (Fig 4C) of at least 200 cells overproducing either Sch9-YFP, Sch9Δ2-402-YFP, Sch9Δ403-824-YFP or the YFP control. Indeed, the C-terminal construct led to the most prominent cell elongation (the highest major/minor diameter ratio), while the cells overproducing the Sch9Δ403-824-YFP construct were indistinguishable from the control ones overproducing YFP (Fig 4B). Thus, cell elongation upon Sch9 overproduction is mediated by the C-terminal part of the protein, which contains its kinase domains.

To find out whether this effect was TOR-dependent, we treated the cells with rapamycin, an antibiotic blocking TORC1 [41]. This treatment led to rounding of cells and abolished the effect of Sch9-YFP, as cells overproducing YFP and Sch9-YFP looked similar (S4A Fig). Thus, the cell elongation we observed upon Sch9 overproduction might be TOR-dependent, as it was dominated by the effect of rapamycin treatment.

To further delve into the mechanism behind cell elongation, we measured bud sizes for the cells and explored the relationship between relative bud size and cell elongation. Relative bud size, which can be used as a parameter characterizing the position of the cell in the cell cycle [40], was determined as the ratio of major diameters of the daughter and mother cells (Fig 4C). This analysis was performed for the control construct (YFP) and the Sch9 allele that had the most pronounced effect (Sch9Δ2-402-YFP). While in the control cell population cell shape (ratio of major/minor diameters of the cell) does not depend on bud size (slope = -0.04, p = 0.46; Fig 4D), when Sch9Δ2-402-YFP is overproduced, cell elongation and bud size have a clear positive relationship (slope = 0.24, p = 0.003; Fig 4D). Then we performed the same analysis for YFP overproduction *vs.* an empty vector control and found no difference (S4B and S4C Fig). In addition, we compared distributions of cells with different bud sizes between Sch9Δ2-402-YFP overproduction and control (YFP overproduction) and found that the distributions were different (p = 0.0009 in Kolmogorov-Smirnov test) with more cells with large buds in the case of Sch9-Δ2-402-YFP overproduction. So, cell elongation caused by Sch9 overproduction is correlated with bud size.

## Discussion

In this work, we show that the Sch9 protein forms detergent-resistant (*i.e.*, amyloid-like) aggregates upon overproduction in yeast cells (Fig 2, S2 Fig). Intriguingly, it seems that both the N-terminal and the C-terminal halves of the protein contain some determinants of aggregation, even though aggregation-prone regions were predicted only in the N-terminal part. This is the distinctive feature of Sch9 because in most cases aggregation-prone region and functional domain do not overlap [42]. Nevertheless, at least one protein (Rnq1) with multiple regions responsible for aggregation was described earlier [43]. Since the C-terminal part of the protein contains conservative kinase domains [24], our data on Sch9 aggregation might turn out to be relevant for similar proteins in other organisms. Thus, it might be important to consider possibility of protein aggregation when studying the mammalian AGC kinases, which are sometimes overproduced in cancers.

The aggregation of Sch9 probably causes its accumulation in large intracellular structures, which we in our system visualize as fluorescent foci. All constructs formed aggregates resistant to cold SDS treatment, as revealed by protein analysis with Western blotting (Fig 2A and 2B and S2B Fig). However, not in all cases aggregates could be visualized with SDD-AGE, probably because of low protein level in the case of constructs lacking the longest N-terminal protein stretches, namely Δ2-250 and Δ2-402 (S2B Fig, SDS-PAGE). As these short proteins were produced at high level, as checked with a fast method of protein extraction based on alkaline lysis (S2A Fig), we suggest that the N-terminal region of the protein is important for its stability. We faced the same problem when trying to analyze aggregation of full length untagged Sch9 encoded by the chromosomal *SCH9* copy. The protein could be detected by Western blotting only if alkaline lysis procedure was used to prepare the sample. Unfortunately, this method of protein extraction requires boiling in SDS and thus does not allow analysis of detergent resistant aggregates in the probe. Thus, we could not directly check whether the wild-type Sch9 forms aggregates. Nevertheless, we suppose that the untagged protein also can aggregate, as there are no obvious limitations. The problem of low-copy Sch9 visualization also did not allow us to check if its aggregates possess prion-like properties, *i.e.*, are self-propagated even after the end of overproduction. This question is very interesting especially in the light of a recent work that revealed that overproduction of different yeast proteins leads to appearance of new phenotypic traits [44]. Thus, from this point of view it is very important to investigate changes of the level of Sch9 upon different treatments. So far, it has been shown that at least carbon source may affect this parameter [2].

Interestingly, for almost all constructs, we noted within-clone variability of fluorescent phenotypes, from multiple small dots to one large dot with possible combinations in between (Fig 1, S2C Fig and Fig 4A). As we could not sort cells prior to protein extraction, we cannot determine which type or types of foci corresponds to detergent resistant aggregates. However, lack of some regions led to visible changes in shape of fluorescent foci. We noticed that constructs lacking one or both C2 domains, which are important for protein-lipid interaction [32], tend to form aggregates of irregular shape (S2C Fig). This result could suggests a link between Sch9 aggregation and its location in the vacuolar membrane, but we did not see colocalization even between round aggregates vacuolar membrane (Fig 3A). Furthermore, these aggregates did not also correspond to IPOD, the structure in which many aggregation-prone proteins are stored in the yeast cell (Fig 3B); thus, we could not identify the subcellular compartment, in which these aggregates are located. It might be possible that overproduced Sch9 forms a specific intracellular structure.

Formation of protein aggregates or phase separated particles may be implicated into different processes [45, 46]. Such examples have been accumulating extensively during past decade. Although the first examples were basically described as different kinds of misfolded protein deposits [47], recent findings demonstrate more and more complicated functions of such complexes. For instance, aggregation of the Whi3 protein in yeast cells acts as a mnemon and changes cellular behavior [48]). In addition, a set of constitutive and likely functional yeast amyloids has been replenished by new examples [49]. Moreover, activation of T cell receptors has been recently shown to lead to formation of liquid droplets enriched by kinases in a model system. These complexes promote actin filament assembly [50]. Following these numerous examples, we can speculate that Sch9 complexes also are implicated in some regulatory processes.

We also found a phenotype connected with *SCH9* overexpression, elongation of cells. Interestingly, even though large cell phenotype was shown for *SCH9* overexpression [2], changes in cell shape, to the extent of our knowledge, has not been reported yet. This difference might be explained by the fact that we exploited a system with high-level constitutive overexpression driven by the *GPD* (*TDH3*) promoter. Here we showed that Sch9-YFP overproduction led to cell elongation, and this effect was more pronounced for cells with larger buds (Fig 4D). We also noted that Sch9-YFP overproduction slowed growth rate (S1D Fig). Taken together, these results suggest that cell elongation caused by Sch9 overproduction is correlated with mitotic delay.

Cell elongation was likely strain specific, as this effect was observed in BY4742 cells but was not so prominent in *sch9Δ*-BY4741 cells (Fig 1). These two strains are closely related and should differ by only a handful of genetic markers: mating type, *LYS2* and *MET15* alleles and of course the presence of *SCH9*. However, the feature determining this phenotypic effect is not either, as there are other BY4741-based strains that react to Sch9 overproduction with clearly seen elongation and at least one *SCH9* strain not closely related to S288C that does not elongate in response to Sch9 overproduction (data not shown). Thus, the mechanism underlying strain specificity of this trait is still to be uncovered.

Our findings for the first time demonstrate the ability of the Sch9 protein to form specific intracellular structures, at least some of which possess amyloid-like properties. In addition, here we attempt to separately analyze functions of the different Sch9 domains and reveal specific effect of the C-terminal region overproduction on cell elongation and existing of several aggregation-prone regions in different parts of the protein. Even though this analysis may be considered as incomplete, we believe that the obtained results complement prior knowledge about Sch9 functions and create a basis for further investigation. Finally, the constructs we present can be exploited to create superior yeast-based model systems to study processes behind AGC kinase overproduction in cancers. These potential model systems could also be useful for testing novel inhibitors of AGC kinases, for example p70S6K, inhibitors of which are already being developed [51–53].

## Materials and methods

### Microbial strains and cultivation procedures

Throughout this work, two S288C-related *S. cerevisiae* strains were used: an *sch9Δ* strain JW 03 038 BY4741 (genotype *MAT*a *his3Δ1 leu2Δ0 met15Δ0 ura3Δ0 sch9Δ::NATMX4* [9, 54]), which is also referred to in the text as *sch9Δ*-BY4741, and *SCH9* strain BY4742 (*MATα his3Δ1 leu2Δ0 lys2Δ0 ura3Δ0* [36]).

*Escherichia coli* strain DH5α [55] was used for plasmid selection, maintenance and amplification. Standard yeast and bacterial media with minor modifications were used [56, 57]. The Gal/Raff medium contained 2% galactose and 1% raffinose instead of glucose. For curing of the [*PIN*^+^] prion, guanidinium chloride was added into the YEPD medium in the final concentration of 5 mM, and cells were passaged three times. Rapamycin treatment was performed with 100 nM rapamycin in liquid SC-Ura medium for 2 hours. Yeast strains were grown at 30 °C and the *E. coli* strain was grown at 37 °C.

### Plasmid construction

Plasmids used in this work and primers used for their construction are listed in S1 and S2 Tables, respectively. Cloning was performed in accordance with standard protocols [57].

To construct p426GPD-SCH9YFP, the *SCH9* ORF without the stop codon was amplified with primers Sch9-F-SpeI and Sch9-R-BamHI and inserted into BamHI/SpeI cut p426GPDSWI1YFP [58]. p426GPD-YFP was constructed by blunting the ends of BamHI/SpeI cut p426GPDSWI1YFP with Klenow fragment and subsequent ligation. p426GPD-SCH9Δ183-256-YFP was made with NheI/XmaJI restriction of p426GPD-SCH9YFP and subsequent vector self-ligation. 426GPD-SCH9Δ2-250-YFP was made from p426GPD-SCH9YFP via obtaining long PCR product and recombination in bacteria [59]. The same method was applied to obtain p426GPD-SCH9Δ91-138Δ183-256-YFP, but in this case p426GPD-SCH9Δ183-256-YFP was used as a template. p426GPD-SCH9Δ2-403-YFP and p426GPD-SCH9Δ403-824-YFP were constructed by replacing the full length *SCH9* with its shorter alleles (obtained as PCR products with the same template) at SpeI/BamHI sites. pRS416-SCH9YFP was constructed by subcloning the XmaJI/KpnI restriction fragment of p426GPD-SCH9YFP into pJU675 [1]. All constructs were verified with restriction digest and insert sequencing. Plasmid maps are available in S1 Maps.

pRS415CUP-RNQ1GFP (Derkatch, unpublished) was used to monitor the [*PIN*^+^] status of the strain, and pAG415GPD-Hsp42-mCherry [39] was used to locate IPOD. pRS416 [60] and pRS426 [61] were used as empty vector controls. Maps of pRS plasmids were modified according to the published corrections [62].

### Microscopy

Staining with FM4-64 (Invitrogen) was performed according to the published protocol [63] with slight modifications: YEPD was used instead of YES, and cells were grown for 120 minutes after washing off non-bound dye. Cells producing YFP or mCherry fusion proteins were grown in synthetic media until late logarithmic phase (cell density about 10^7^ cells/ml), mixed with glycerol (25% final concentration) and observed with Zeiss Axio Scope.A1. The following filters were used: 46 (excitation peak 500 nm / emission peak 535 nm) for Sch9-YFP fusions, 63 HE mRFP shift free (excitation peak 572 nm / emission peak 629 nm) for the Hsp42-mCherry fusion and FM4-64, 74HE GFP+mRFP shift free for detection of autofluorescence or simultaneous detection of YFP and mCherry (excitation peaks 483 and 569 nm / emission peaks 636 nm). Shooting exposure was chosen empirically for informativeness and may not necessarily be the same for different constructs.

### Biochemical methods

For protein extraction, cells were grown in synthetic media until late logarithmic phase (cell density about 10^7^ cells/ml) and collected with centrifugation (about 10^7^ cells for alkaline lysis [64] or about 2×10^8^ cells for mechanical cell disruption [65]). As alkaline lysis allows to achieve higher protein concentration [64] and reduced degradation (compare S2A and S2B Fig), but due to the procedure requires sample boiling, it was used to check protein production, while mechanical cell disruption was used to assess protein aggregation.

SDS-PAGE [57] or SDD-AGE [27] was used for separation of the proteins, and PVDF membranes (GE Healthcare) were used for semi-dry [57] or capillary transfer [66], respectively. Blots were probed with either anti-732-743-Sch9 and anti-phospho-Thr737-Sch9 [67] or anti-Tag(CGY)FP (Evrogen AB121) antibodies and photographed with GeneGnome (SynGene).

### Data analysis

The ArchCandy [28] program was used for prediction of amyloidogenic regions (0.575 was used as threshold value without any additional built-in filters). Following the recommendation of the developers, we considered only those β-arches that were located in unstructured regions. The IUPred program [68] with *long* option was used for prediction of such regions, the recommended threshold 0.5 was used.

ImageJ [69] was used for measuring major and minor axes of cells with the *fit ellipse* measurement option. Custom R [70] scripts were also used for data analysis. The Mann-Whitney U test implemented in the coin package [71] was used to test differences in ellipse axis ratio. Distribution of cells with buds of different sizes were compared with the ks.test function, and linear regression models were built with the lm function of the base R package [70]. The ggplot2 package [72] was used to plot scatterplots with regression lines and 95% confidence intervals.

## Supporting information

S1 Fig Sch9-YFP is functional and not toxic when overproduced. A: Western blot probed with anti-732-743-Sch9 antibody. B: Western blots probed with anti-732-743-Sch9 or anti-phospho-Thr737-Sch9 antibody. The same lysates were loaded into both gels. Approximate molecular weight in kDa is shown according to a standard protein weight ladder. C: Five-fold serial dilutions of the respective transformants. A-C: *WT* and *Δ* designate BY4742 and *sch9Δ*-BY4741 strains, respectively. Dashed lines mark additional lanes removed for clarity. SCH9-YFP, p426GPD-SCH9YFP; YFP, p426GPD-YFP. D: Five-fold serial dilutions of representative transformants of the *sch9Δ*-BY4741 strain. Plasmids used (from left to right): pRS416, pJU675, pRS416-SCH9YFP, p426GPD-YFP, p426GPD-SCH9YFP. C-D: Cell concentration decreases from top to bottom.
(TIF)

S2 Fig Representative examples of results of the amyloidogenicity analysis of different Sch9 deletion constructs. A: Western blotting of membranes with boiled cell lysates obtained with alkaline lysis and separated with SDS-PAGE. The primary antibodies used for probing are shown under each blot. B: Results of fluorescent microscopy, SDD-AGE and SDS-PAGE analysis of cells overproducing each Sch9 construct (shown in the leftmost column). Dashed lines separated different fields of view chosen from the same slide or different lanes from the same blot. Lysates of cells overproducing the full-length protein (WT) are shown for comparison on each blot image. The plus and minus signs indicate whether the sample was boiled. Scale bars on microphotographs correspond to 5 μm. Numbers to the left of blots show the position of the corresponding protein molecular weight standard (kDa).
(TIF)

S3 Fig Sch9-YFP fluorescent patterns are independent of the [*PIN*^+^] prion status. Dashed lines separated different fields of view chosen from the same slide. The scale bar corresponds to 5 μm. BF, bright-field microscopy. For Rnq1-GFP overproduction, CuSO_4_ was added to the final concentration of 50 μM, and then cells were incubated for 3 hours.
(TIF)

S4 Fig YFP overproduction does not affect cell shape, while rapamycin treatment overcomes the effect of Sch9-YFP overproduction. A: Microphotographs of cells treated with rapamycin. Dashed lines separated different fields of view from the same slide. The scale bar indicates 8 μm. B: Box plot summarizing major / minor cell diameter ratios of at least 90 cells with each construct shown below the graph. Each dot corresponds to individual cell, the central line is the median, box edges show the interquartile range, and whisker length correspond to maximum or minimal values within 1.5 interquartile ranges up and down from the box. *n.s.*, p > 0.05 in Mann-Whitney test. C: Scatterplot visualizing the relationship between relative bud length and major / minor mother diameter ratio.
(TIF)

S1 Table List of plasmids used in this work. The description column lists the characteristics in the following order: *S. cerevisiae* replication origin, *S. cerevisiae* selective marker gene, *E. coli* selective marker gene, promoter, and gene of interest.
(XLS)

S2 Table List of primers used in this work. (XLS)

S1 Maps Maps of the plasmids constructed in this work and other plasmids used (if available). (ZIP)
