# Supplementary figures and images for: Overproduction of Sch9 leads to its aggregation and cell elongation in Saccharomyces cerevisiae
Source: PLoS One. 2018 Mar 1;13(3):e0193726. doi: 10.1371/journal.pone.0193726 (PMC5832320; doi:10.1371/journal.pone.0193726)

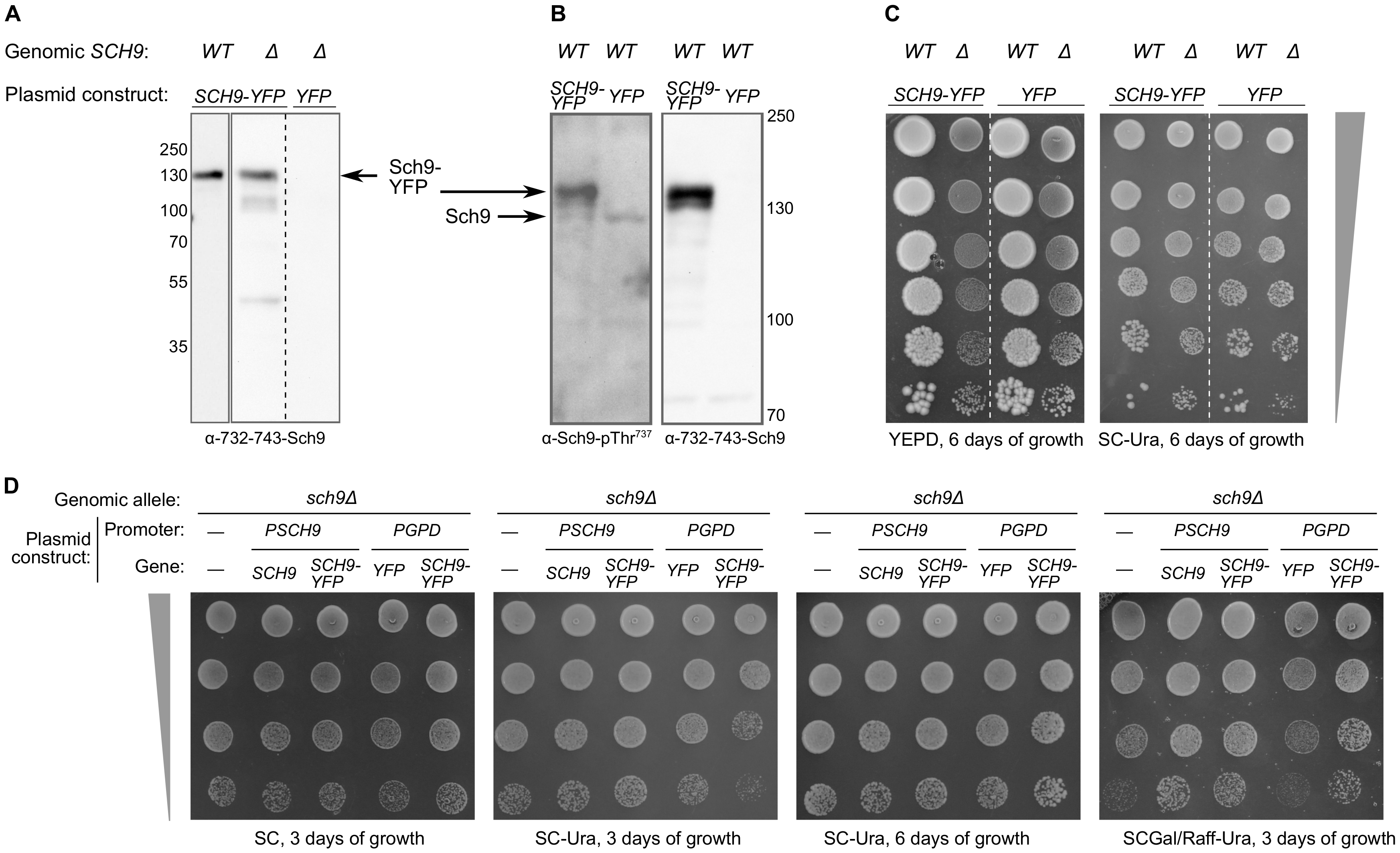

Supplement: S1 Fig — A: Western blot probed with anti-732-743-Sch9 antibody. B: Western blots probed with anti-732-743-Sch9 or anti-phospho-Thr737-Sch9 antibody. The same lysates were loaded into both gels. Approximate molecular weight in kDa is shown according to a standard protein weight ladder. C: Five-fold serial dilutions of the respective transformants. A-C: WT and Δ designate BY4742 and sch9Δ-BY4741 strains, respectively. Dashed lines mark additional lanes removed for clarity. SCH9-YFP, p426GPD-SCH9YFP; YFP, p426GPD-YFP. D: Five-fold serial dilutions of representative transformants of the sch9Δ-BY4741 strain. Plasmids used (from left to right): pRS416, pJU675, pRS416-SCH9YFP, p426GPD-YFP, p426GPD-SCH9YFP. C-D: Cell concentration decreases from top to bottom. (TIF) [file pone.0193726.s001.tif]

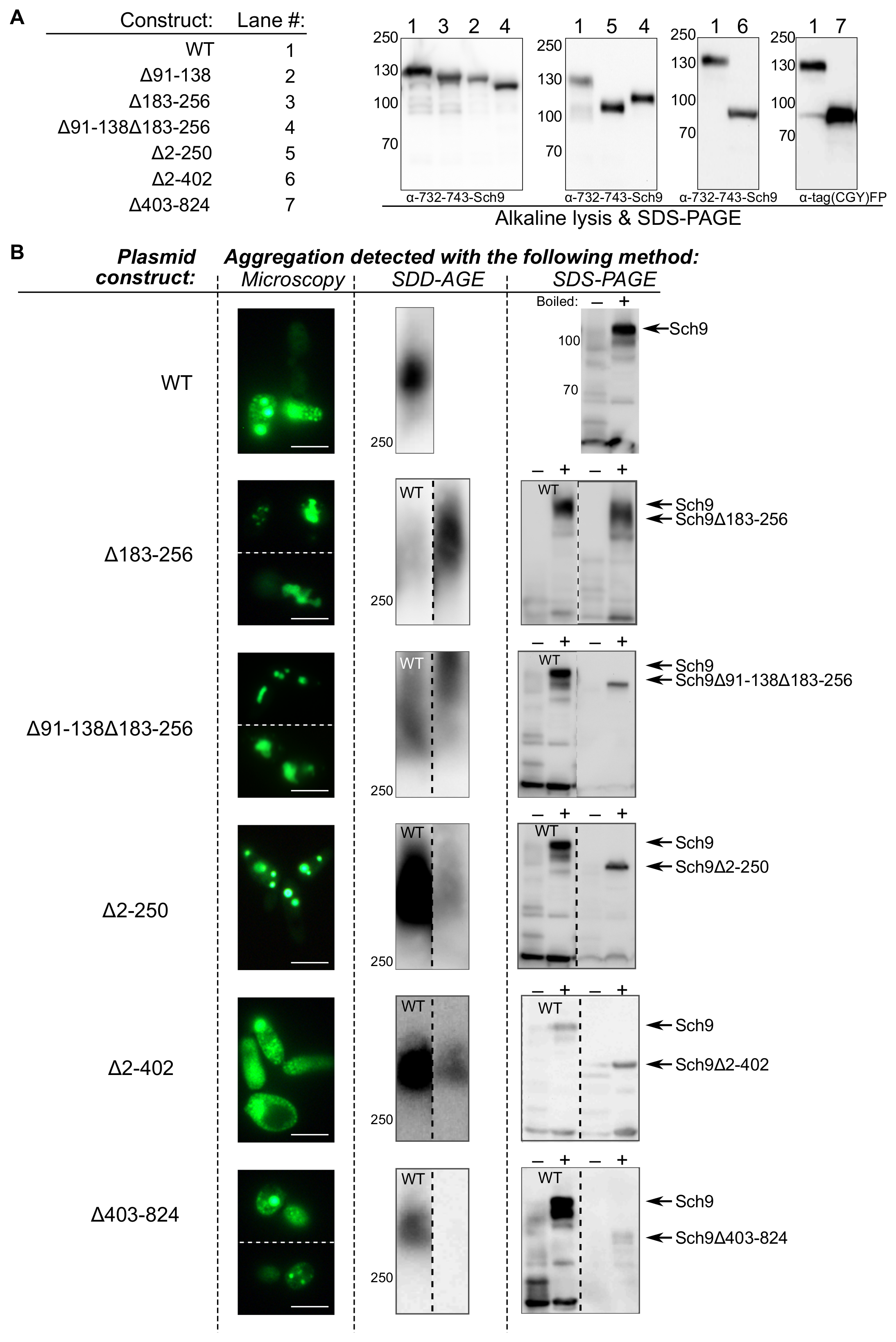

Supplement: S2 Fig — A: Western blotting of membranes with boiled cell lysates obtained with alkaline lysis and separated with SDS-PAGE. The primary antibodies used for probing are shown under each blot. B: Results of fluorescent microscopy, SDD-AGE and SDS-PAGE analysis of cells overproducing each Sch9 construct (shown in the leftmost column). Dashed lines separated different fields of view chosen from the same slide or different lanes from the same blot. Lysates of cells overproducing the full-length protein (WT) are shown for comparison on each blot image. The plus and minus signs indicate whether the sample was boiled. Scale bars on microphotographs correspond to 5 μm. Numbers to the left of blots show the position of the corresponding protein molecular weight standard (kDa). (TIF) [file pone.0193726.s002.tif]

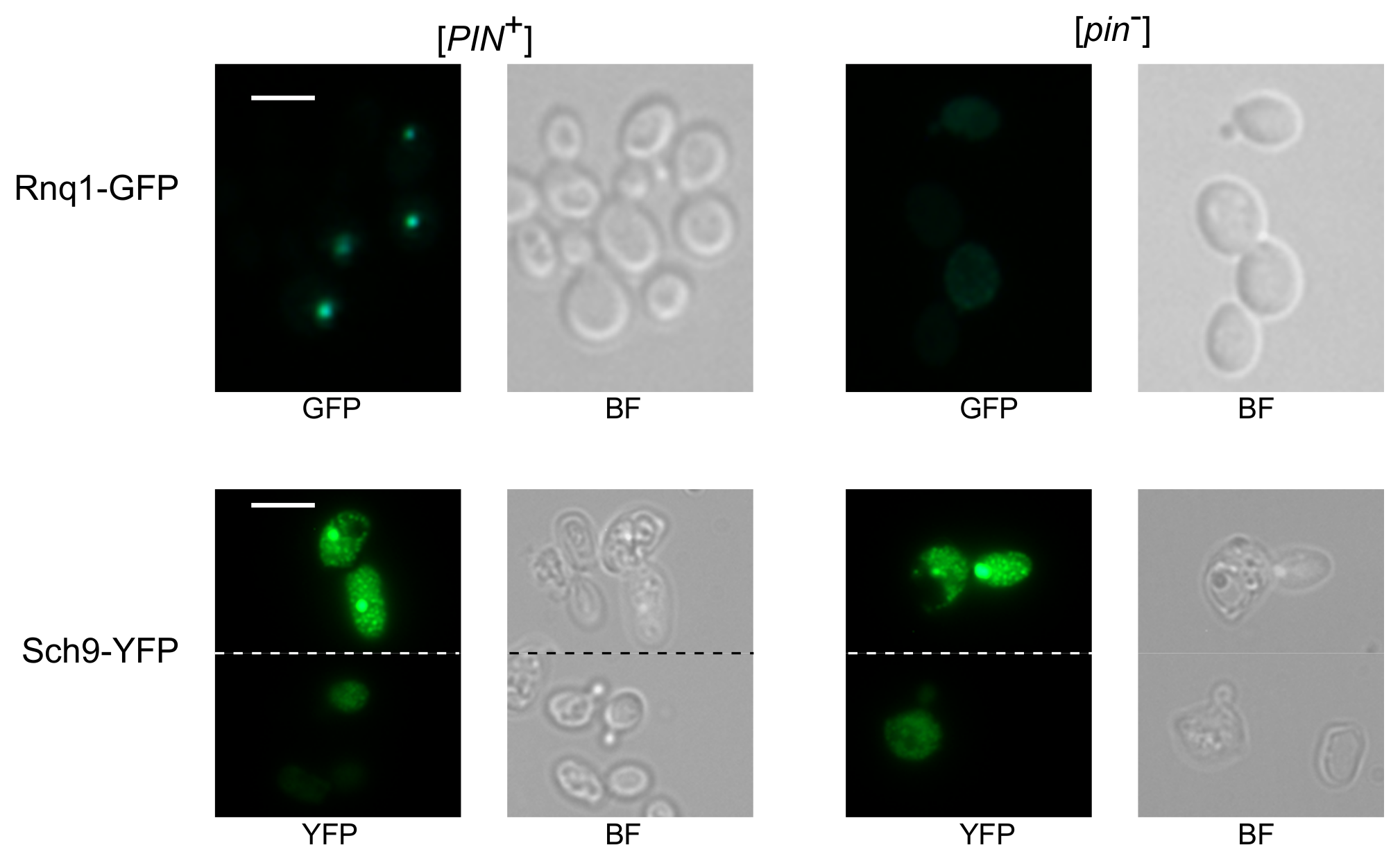

Supplement: S3 Fig — Dashed lines separated different fields of view chosen from the same slide. The scale bar corresponds to 5 μm. BF, bright-field microscopy. For Rnq1-GFP overproduction, CuSO4 was added to the final concentration of 50 μM, and then cells were incubated for 3 hours. (TIF) [file pone.0193726.s003.tif]

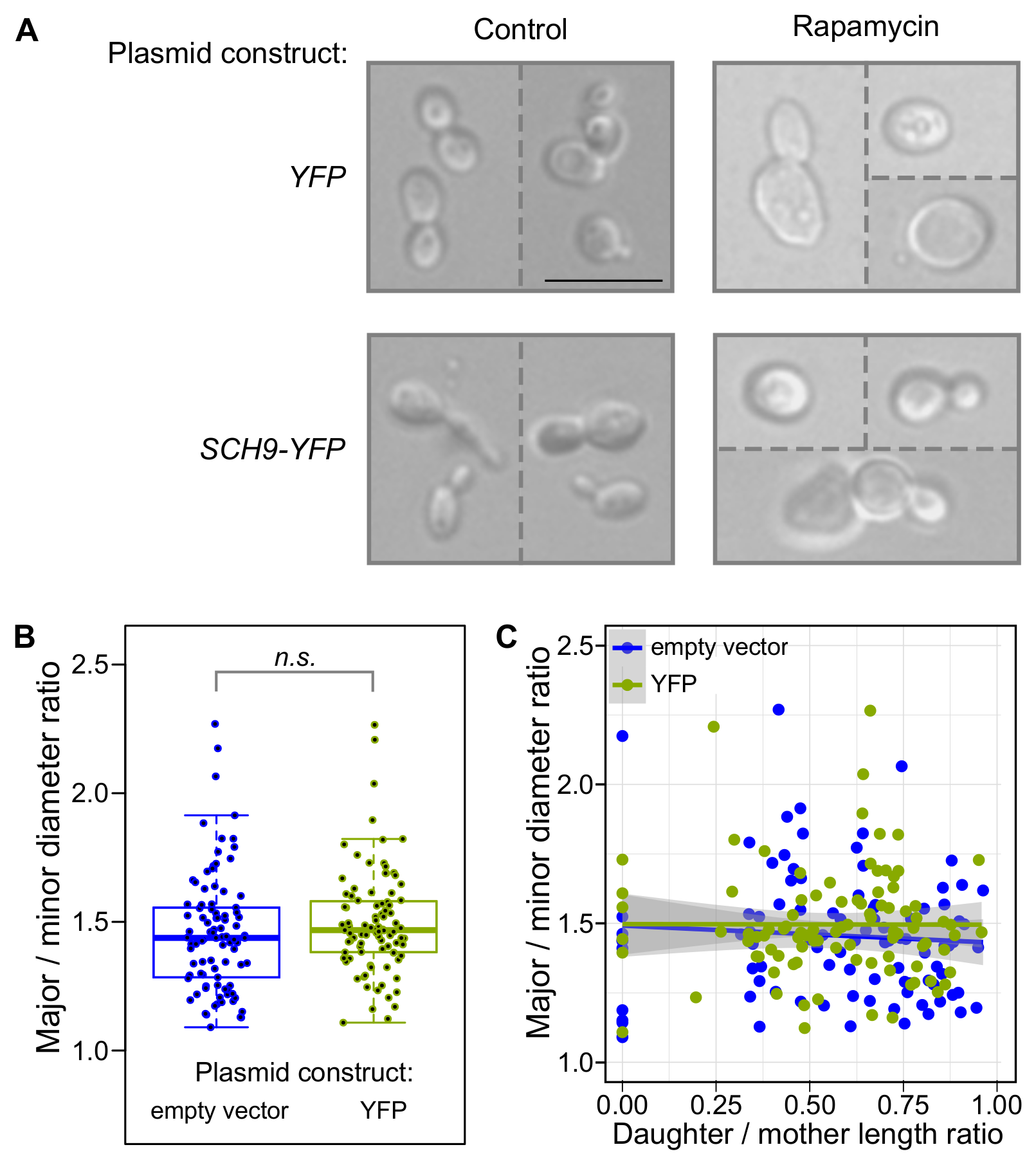

Supplement: S4 Fig — A: Microphotographs of cells treated with rapamycin. Dashed lines separated different fields of view from the same slide. The scale bar indicates 8 μm. B: Box plot summarizing major / minor cell diameter ratios of at least 90 cells with each construct shown below the graph. Each dot corresponds to individual cell, the central line is the median, box edges show the interquartile range, and whisker length correspond to maximum or minimal values within 1.5 interquartile ranges up and down from the box. n.s., p > 0.05 in Mann-Whitney test. C: Scatterplot visualizing the relationship between relative bud length and major / minor mother diameter ratio. (TIF) [file pone.0193726.s004.tif]
